# Outcomes and Prognostic Factors in Tumor-Related Amputations: A Retrospective Cohort Study of 132 Cases over Two Decades

**DOI:** 10.3390/jcm15031293

**Published:** 2026-02-06

**Authors:** Sebastian Breden, Maximilian Stephan, Florian Hinterwimmer, Sarah Consalvo, Ulrich Lenze, Rüdiger von Eisenhart-Rothe, Carolin Knebel

**Affiliations:** Department of Orthopaedics and Sports Orthopaedics, TUM Universitätsklinikum Rechts der Isar, Ismaninger Str. 22, 81675 Munich, Germany

**Keywords:** musculoskeletal tumors, sarcoma, amputation, orthopedic oncology

## Abstract

**Background**: Despite advancements in limb-sparing surgery (LSS), adjuvant therapies, and imaging techniques, amputations remain necessary in certain cases, including locally advanced tumors, inadequate resections, or palliative scenarios. This study aims to provide an overview of tumor-related amputations, comparing primary and secondary amputations in terms of survival, recurrence, and surgical outcomes. **Methods**: A retrospective cohort study of 132 patients undergoing tumor-related amputations between 2004 and 2023 at a tertiary care center was conducted. Patients were stratified by amputation level (major vs. hand/foot) and timing (primary vs. secondary). Kaplan–Meier survival and multivariate regression analysis identified prognostic factors. **Results**: Major amputations accounted for 77% of cases, while 23% involved the hands or feet. Primary amputations constituted 55% of procedures, and 45% were secondary interventions. Overall survival was 123 months (95% CI, 105–142), with a 5-year survival rate of 66% and a 10-year survival rate of 53%, respectively. Hand/foot amputations showed superior survival compared to major amputations (*p* = 0.032). Local recurrence emerged as the only significant predictor of overall survival (*p* = 0.033). **Conclusions**: Tumor-related amputations remain crucial in musculoskeletal oncology. Survival outcomes are comparable between primary and secondary amputations, but hand/foot amputations are associated with improved survival. Achieving local control is critical, underscoring the need for precise surgical planning.

## 1. Introduction

For many years, amputation was the only treatment option for musculoskeletal tumors of the extremities [1]. It was not until the groundbreaking work of Rosenberg et al. in 1982 that limb-sparing surgery (LSS) became a viable alternative for the management of sarcomas [2]. In the following decades, advancements in (neo-)adjuvant therapies [3,4], improvements in surgical techniques [5,6] and innovations in radiological imaging [7,8] established LSS as the primary mode of surgical treatment for extremity sarcomas.

Despite these advancements, amputation remains necessary in certain cases [9,10,11]. Common indications include locally advanced tumors in which the size of the lesion or infiltration of critical structures precludes LSS [8,10,12], as well as extensive contamination after inadequate resections or pathological fractures [13,14]. Additionally, amputations play a role in palliative settings, such as metastatic disease from various primaries, in which shorter recovery times are prioritized over limb preservation [10,15].

A key question remains whether attempting LSS in borderline cases is preferable to opting for primary amputation, particularly given that recent studies have reported shortened survival times for amputees compared to patients receiving LSS [16,17]. However, retrospective analyses of these groups may lead to misleading conclusions, as patients undergoing amputation are often in more advanced stages of cancer than those whose limbs could be spared [17].

In daily clinical practice, the decision between limb-sparing surgery and amputation is rarely binary. Particularly in borderline cases, surgical planning must balance oncological safety, functional outcome, complication risk, and the patient’s overall prognosis [9,10]. Factors such as tumor size, involvement of neurovascular structures, previous unplanned resections, and anticipated surgical morbidity often complicate decision-making. In this context, multidisciplinary tumor boards play a central role in integrating oncological, surgical, radiological, and patient-related factors to determine the most appropriate treatment strategy [18]. Nevertheless, evidence guiding these complex decisions remains limited, especially with regard to the timing of amputation and its impact on long-term outcomes. A potentially better approach to evaluating the appropriateness of LSS in borderline cases would be to compare outcomes between secondary amputations following failed LSS and primary amputations performed as first-line treatment. Evidence directly comparing primary and secondary (delayed) amputations remains limited. Erstad et al. reported no significant difference in survival between early and delayed amputation for extremity sarcoma, and more recent retrospective data have similarly suggested comparable oncological outcomes after secondary amputation following failed limb-sparing surgery [10,19].

Nevertheless, the risk of inadequate resection is higher in challenging LSS procedures compared to amputations [17]. This is clinically relevant, as incomplete resections in some sarcoma types have been associated with higher rates of recurrence and tumor-related mortality [20,21].

In this study, we aim to provide a comprehensive overview of all oncological amputations performed at our institution and to compare primary and secondary amputations with regard to overall and event-free survival, as well as local recurrence and revision surgery rates.

## 2. Materials and Methods

### 2.1. Ethical Approval

Ethical approval for this study was obtained from the Ethics Committee of the School of Health and Medicine, Technical University of Munich (Approval N 2024-342-S-CB). All included patients had provided broad consent for the use of their data for research purposes. Given the retrospective design and the pseudonymized nature of the dataset, the requirement for written informed consent was waived.

### 2.2. Study Design and Patient Selection

This retrospective cohort study was conducted at a tertiary referral center specializing in musculoskeletal oncology. All patients who underwent an amputation for oncological reasons or due to complications following local tumor treatment between 2004 and 2023 were screened for eligibility.

Inclusion criteria comprised patients with primary malignant bone tumors, soft-tissue sarcomas, as well as patients undergoing amputation for metastatic disease or tumor-related complications. Patients were excluded if essential clinical or follow-up data were unavailable or incomplete, precluding reliable outcome assessment.

### 2.3. Data Collection

Demographic, clinical, radiological, surgical, and histopathological data were extracted from institutional electronic medical records. Collected variables included age, sex, tumor entity, grading and staging, tumor size, anatomical location, indication for amputation, amputation level, resection margins, and perioperative complications.

Follow-up data were obtained through scheduled outpatient visits and, when necessary, supplemented by structured telephone interviews. Survival status, local recurrence, systemic disease progression, revision surgery, and cause of death were recorded.

Of the initially identified patients, a subset was excluded from the final analysis due to insufficient data completeness. These exclusions predominantly concerned secondary amputations in which preceding surgical procedures had been performed outside our institution, resulting in incomplete documentation of prior treatments, tumor status, or perioperative details. In addition, amputations that were performed externally and subsequently referred to our center were excluded. Consequently, only patients with complete documentation of the amputation procedure and adequate clinical follow-up were included in the final cohort.

### 2.4. Patient Stratification and Treatment Decision-Making

Patients were stratified according to the level of amputation into major amputations and amputations involving the hand or foot. Each group was further subdivided into patients undergoing primary amputation as the initial surgical treatment and those requiring secondary amputation following failed limb-sparing surgery.

All treatment decisions, including the indication for amputation, were made within a multidisciplinary tumor board setting involving orthopedic oncologists, medical oncologists, radiologists, pathologists, and radiation oncologists. The documented rationale for amputation—such as tumor extent, involvement of neurovascular structures, contamination after prior surgery, or palliative intent—was recorded for each patient.

### 2.5. Outcome Definitions

Overall survival (OS) was defined as the time from amputation to death from any cause or last follow-up. Patients alive at last follow-up were censored.

Local recurrence was defined as tumor recurrence at or adjacent to the amputation site. In all cases, recurrence was histologically confirmed, either by pathological examination following surgical re-resection or, in patients treated with palliative intent, at autopsy.

Revision surgery was defined as any unplanned surgical intervention performed after the initial amputation procedure. Planned staged procedures that were part of the intended treatment course, such as delayed wound closure or scheduled skin grafting, were explicitly excluded from this definition.

Revision surgeries were categorized according to their primary indication, including deep or superficial surgical site infection, skip metastases or tumor recurrence. All revision events and their indications were extracted from institutional operative reports and postoperative documentation and were verified during data curation.

### 2.6. Statistical Analysis

Statistical analyses were performed using SPSS version 29 (SPSS Inc., Chicago, IL, USA). Continuous variables are presented as means with ranges and were compared using Student’s *t*-test or the Mann–Whitney U test, depending on data distribution. Categorical variables were analyzed using chi-square tests.

Survival analyses were conducted using the Kaplan–Meier method, with group comparisons performed using the Log-Rank test. Prognostic factors for overall survival and local recurrence were assessed using univariate and multivariate Cox proportional hazards regression models. Age and tumor size were entered as continuous variables. Proportional hazards assumptions were assessed and not violated. A two-sided *p* value of <0.05 was considered statistically significant.

## 3. Results

The initial database search identified 145 tumor-related amputations performed at our center between 2004 and 2023. After excluding patients with insufficient data concerning surgeries performed outside of our center, 132 cases were included in the study.

The mean age at the time of amputation was 51 years (range, 4–92 years). The cohort consisted of 80 males and 52 females, resulting in a male-to-female ratio of 1.5:1. Major amputations accounted for 102 cases (77%), while 30 amputations (23%) involved the hands or feet. Of all amputations, 73 (55%) were primary interventions and 59 (45%) were secondary procedures. This division resulted in four distinct cohorts, as shown in Table 1.

There were no significant differences in age or sex between the cohorts (*p* = 0.861 and *p* = 0.505). Overall, the majority of tumors were primary cancers originating from soft tissues (60; 45%) or bones (48; 36%). Fewer amputations were performed due to metastases (13; 10%) or dermatological tumors (11; 8%). Table 1 provides a breakdown of the cohorts.

### 3.1. Amputations of Hands and Feet

In 30 cases, amputations were performed at the hands or feet. Of these, 16 procedures (53%) were primary amputations (Cohort 1; C1) and 14 (47%) were secondary amputations (Cohort 2; C2). Twenty cases (67%) involved primary musculoskeletal malignancies, while 10 (33%) involved metastases or skin cancers. Compared to major amputations, a significantly higher proportion of skin tumors was observed in this group (*p* < 0.0001). In six cases, benign tumors led to amputations of toes or fingers. Tumors were located in the hands in four cases (13%) and in the feet in 26 cases (87%). Tumor-specific characteristics are summarized in Table 2.

Most amputations (27/30; 90%) were performed due to the unique unicompartmental anatomy of the hands and feet. In 13 cases (93% of secondary amputations), prior inadequate resections performed outside our center necessitated secondary amputation. In patients undergoing secondary amputations of the hand or foot, the mean time interval between the initial limb-sparing treatment and secondary amputation was 62 months (range: 21 days to 423 months). The levels of amputation for all cases are shown in Table 3.

Additional treatments included neoadjuvant chemotherapy (2 cases), neoadjuvant radiotherapy (4 cases), and targeted therapy (2 cases). Adjuvant therapies included four courses of chemotherapy, seven radiotherapies targeting the lymphatic drainage area, and three targeted therapies. R0 resection margins were achieved in all but one secondary amputation (R1).

Local recurrence occurred in one case of completely resected (R0) extra-skeletal myxoid chondrosarcoma after 188 months, requiring unplanned follow-up surgery. In this cohort, only one unplanned follow-up surgery was necessary for a locally recurring tumor (see Table 4).

In the primary amputation cohort (C1), one tumor-related death was recorded; in the secondary cohort (C2), four tumor-related deaths occurred. The mean follow-up was 69 months (range, 20–193) in C1 and 45 months (range, 7–115) in C2.

### 3.2. Major Amputations

A total of 102 major amputations were performed: 57 (56%) were primary interventions (Cohort 3; C3), and 45 (44%) followed failed limb-sparing surgery (Cohort 4; C4). In 88 cases (86%), the tumors were primary musculoskeletal malignancies, and 14 cases (14%) were metastases, with two originating from skin cancer. No benign tumors or primary skin cancers led to major amputations. The proportion of metastases was significantly higher in major amputations compared to hand or foot amputations (*p* < 0.001). Tumor-specific characteristics are detailed in Table 2.

The main reasons for primary major amputations included tumor size (19; 33%), infiltration of vital structures such as vessels or nerves (18; 31%), and ulceration or joint invasion (5; 9%). Less common reasons included contamination due to fractures or of the foot (3 cases each; 5%). Rare indications included multiple metastases, extensive hematomas, or palliative indications. Indications for major amputations are summarized in Table 5.

Secondary major amputations followed 19 interventions (42%) performed outside our center, of which 14 (74%) involved inadequate resections requiring oncological completion. In 25 cases, primary LSS was performed at our center, achieving free margins (R0) in 20 cases. One secondary amputation was required due to tumor progression following attempted definitive radiotherapy. For secondary major amputations, the mean interval between the initial limb-sparing treatment and amputation was 43 months (range: 30 days to 236 months).

With regard to perioperative oncological treatment, both neoadjuvant and adjuvant therapies were frequently applied in patients undergoing major amputation. In the primary amputation group, neoadjuvant radiotherapy was administered in 22 patients (39%), whereas neoadjuvant chemotherapy was used in 1 patient (2%); other neoadjuvant systemic therapies were applied in 2 patients (4%). Adjuvant radiotherapy represented the most common postoperative treatment and was administered in 27 patients (47%), followed by adjuvant chemotherapy in 2 patients (4%) and other adjuvant systemic therapies in 1 patient (2%).

In the secondary amputation group, neoadjuvant treatments were more commonly applied, including neoadjuvant radiotherapy in 19 patients (42%) and neoadjuvant chemotherapy in 13 patients (29%); other neoadjuvant systemic therapies were used in 2 patients (4%). Adjuvant radiotherapy was administered in 19 patients (42%), while adjuvant chemotherapy was given in 7 patients (16%) and other adjuvant systemic therapies in 2 patients (4%).

Residual or recurrent tumors accounted for the majority of secondary amputations (34 cases; 76%). Indications included tumor size (16; 36%), infiltration of nerves or vessels (6; 14%), ulceration (5; 11%), and contamination of the foot (2; 4%). Surgery-related complications (e.g., infections(6; 14%), hematomas (3; 6%), vascular occlusion (1), inadequate osteosynthesis (1)) accounted for 11 cases (24%).

The levels of major amputations are listed in Table 3. R0 margins were achieved in 56 (98%) primary amputations and 40 (89%) secondary amputations.

Local recurrence occurred in three primary amputations (C3) after a mean of 3.1 months (range, 1.6–6.1 months). These developed after complete resections (R0) of a chondrosarcoma, an osteosarcoma, and an epithelioid sarcoma. In these cases, no further surgical treatments were undertaken due to palliative circumstances.

Fourteen unplanned revision surgeries were required in this cohort, mostly for infections (13 cases), and one second amputation for skip metastases. In C4, seven recurrences were observed after a mean of 7.7 months (range, 3.1–14.8 months), with 12 unplanned surgeries (9 infections, 3 recurrences). See Table 4. The mean follow-up was 44 months for C3 and 58 months for C4.

### 3.3. Local Recurrence and Revision Surgery

Local recurrence rates did not differ significantly between hand/foot and major amputations (*p* = 0.124) or between primary and secondary amputations (*p* = 0.382). No statistical testing was conducted within the hand and foot group due to only one recurrence. Between cohorts 3 and 4, no significant difference in recurrence rates was observed (*p* = 0.295).

Multivariate analysis showed that patient sex, age, cohort, tumor size, grading, entity group, and presence of metastases were not significant predictors of recurrence.

Major amputations had a significantly higher risk of revision surgery compared to hand or foot amputations (*p* = 0.017). No significant correlations were found between C1 vs. C3 (*p* = 0.065) or C2 vs. C4 (*p* = 0.127). Similarly, no significant differences were found between primary and secondary amputations regarding revision surgery (*p* = 0.887). In multivariate analysis, neither demographic nor tumor-related factors were significant predictors for revision surgeries.

### 3.4. Survival

**Figure 1:**
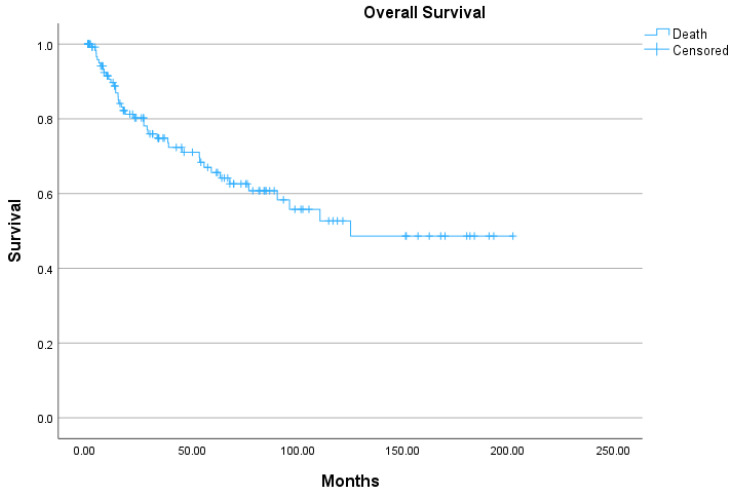
Overall survival, time in months.

Of the 132 patients, 90 (68%) were alive at the time of analysis, 41 (31%) had died from tumor-related causes, and one patient had died from other causes. The mean overall survival was 123 months (95% CI, 105–142 months), with a 5-year survival rate of 66% (95% CI, 63.8–67.3%) and a 10-year survival rate of 53% (95% CI, 51.1–54.3%). The Kaplan–Meier plot is shown in Figure 1.

**Figure 2:**
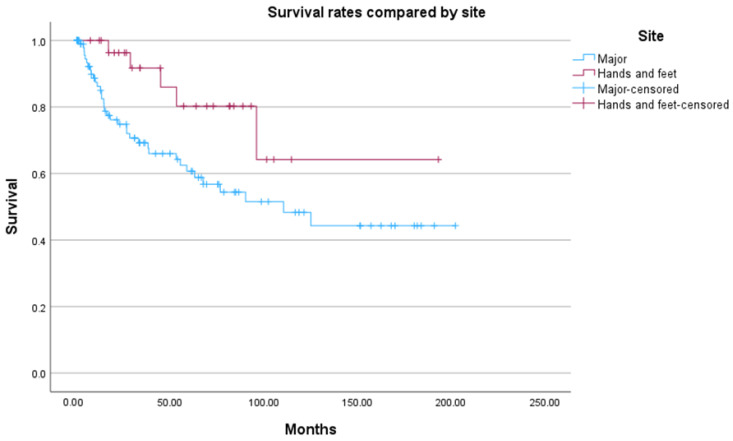
Kaplan–Meier plot comparing major amputations and amputations at the hands and feet. Time in months. Log-Rank Test: *p* = 0.032.

Patients who underwent hand or foot amputations had a mean overall survival of 147 months (95% CI, 111–183 months), compared to 113 months (95% CI, 93–135 months) for major amputations (*p* = 0.032) (Figure 2). Significant survival differences were also observed between the primary cohorts (*p* = 0.015), but not between the secondary cohorts (*p* = 0.694).

**Figure 3:**
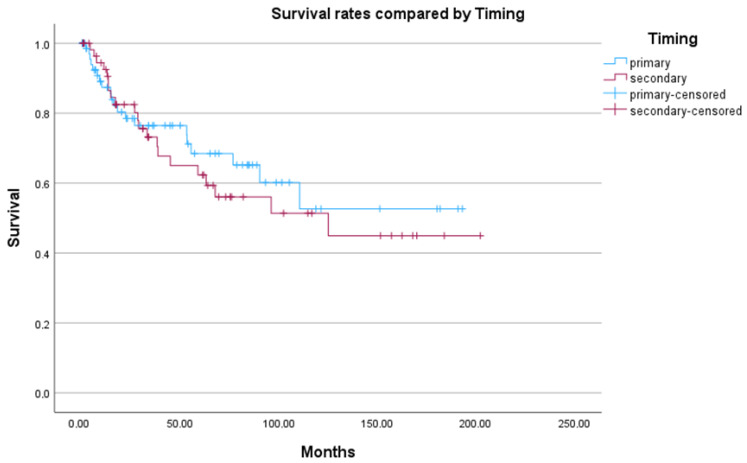
Kaplan–Meier Plot comparing primary versus secondary amputations. Time in months. Log-Rank Test: *p* = 0.608.

No significant difference in overall survival was observed between primary and secondary amputations (*p* = 0.608). Interestingly, primary amputations showed slightly higher mortality in the early years, but after five years, survival favored the primary amputation group (see Figure 3).

**Figure 4:**
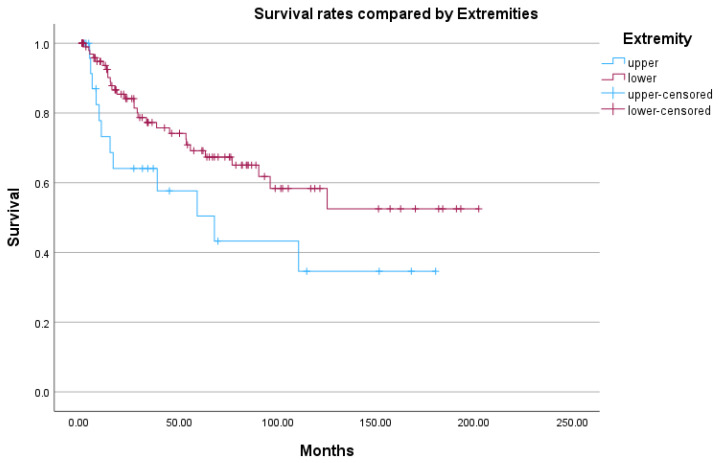
Kaplan–Meier plot comparing upper and lower extremity amputations. Time in Months. Log-Rank Test: *p* = 0.051.

Amputations at the upper limb were associated with shorter survival compared to those at the lower extremity, although this difference was not statistically significant (*p* = 0.051) (Figure 4).

No significant differences were found between the two hand and foot cohorts (*p* = 0.052) or between the two major amputation cohorts (*p* = 0.774).

In multivariable Cox regression analysis, local recurrence was the only independent predictor of overall survival (HR 2.21, 95% CI 1.01–4.85; *p* = 0.048), whereas amputation level, timing of amputation, age, tumor size, and metastatic status were not significantly associated with survival (Table 6).

## 4. Discussion

This study provides a comprehensive analysis of tumor-related amputations performed at a tertiary musculoskeletal tumor center over nearly two decades. Although limb-sparing surgery (LSS) has become the standard of care for extremity sarcomas, our findings demonstrate that amputation continues to play a relevant role in selected clinical scenarios, including advanced local disease, failed LSS, and palliative treatment settings. These results are consistent with previous reports emphasizing that amputation remains an essential component of musculoskeletal oncological surgery despite substantial advances in multimodal treatment strategies [1,10,12].

### 4.1. Patient Demographics and Tumor Characteristics

The predominance of major amputations (77%) observed in our cohort is in line with earlier studies, in which extensive tumor size, aggressive local growth, or involvement of critical neurovascular structures frequently necessitated proximal procedures [10]. The male predominance (male-to-female ratio 1.5:1) likewise reflects previously reported epidemiological patterns in sarcoma populations [10,24].

Soft-tissue sarcomas and primary bone tumors accounted for the majority of amputations in our cohort. Compared to general epidemiological data, the proportion of bone tumors was relatively high, suggesting that primary bone malignancies may more frequently require amputation due to their anatomical location, biological behavior, or limitations of limb-sparing reconstruction [23].

### 4.2. Clinical Implications of Primary Versus Secondary Amputation

A central finding of this study is the absence of a significant difference in overall survival between primary and secondary amputations. This observation is clinically relevant, as it suggests that an initial attempt at limb-sparing surgery does not necessarily compromise long-term survival if secondary amputation becomes necessary. These findings support an individualized and cautious approach in borderline cases, where limb preservation may be considered without automatically accepting an oncological disadvantage should local control ultimately fail.

At the same time, differences in early and late mortality patterns between primary and secondary amputations underline the complexity of treatment sequencing. Higher early mortality after primary amputation likely reflects more advanced disease at presentation, whereas increased late mortality following secondary amputation may be influenced by tumor recurrence, cumulative treatment burden, or complications related to prior limb-sparing procedures. These results reinforce the importance of multidisciplinary decision-making, where oncological safety, functional expectations, and anticipated treatment morbidity must be carefully balanced.

The observed time intervals between initial limb-sparing treatment and secondary amputation, which differed between minor and major amputations, further illustrate that conversion to amputation may represent both early post-surgical complications and late tumor-related morbidity, depending on anatomical site and disease course.

Our findings are consistent with previous retrospective analyses suggesting comparable survival outcomes between early and delayed amputations in sarcoma patients [10,19]. By focusing on primary versus secondary amputations rather than comparing LSS to amputation, this study avoids the substantial selection bias inherent in retrospective comparisons between these fundamentally different patient groups [16,17].

### 4.3. Local Recurrence and Revision Surgery

Local recurrence rates did not differ significantly between primary and secondary amputations or between hand/foot and major amputations. Notably, local recurrence emerged as the only significant predictor of overall survival in multivariate analysis. This finding underscores that, even in the setting of amputation, durable local control remains a key determinant of long-term outcome. Similar associations between local recurrence and survival have been reported in previous sarcoma studies, highlighting the prognostic relevance of local disease control regardless of surgical extent [9,20,21].

Major amputations were associated with a significantly higher risk of revision surgery compared to amputations at the hand or foot, likely reflecting greater surgical complexity and higher rates of wound-related complications. These findings emphasize the importance of meticulous surgical technique, careful perioperative management, and realistic preoperative counseling, particularly in patients undergoing major amputations [12].

### 4.4. Survival Outcomes

Overall survival rates in this cohort—66% at five years and 53% at ten years—are comparable to those reported in previous studies of sarcoma patients undergoing amputation [10,12]. The observed survival advantage in patients undergoing amputations at the hand or foot should be interpreted with caution. From a clinical perspective, this finding is not specific to the post-amputation setting but probably reflects fundamental differences in disease stage, tumor biology, and anatomical constraints between distal and proximal tumors. Distal extremity tumors are more likely to present at earlier stages, allow complete resection with limited surgical extent, and are less frequently associated with extensive neurovascular involvement or systemic disease.

Consequently, amputation level should be regarded as a surrogate marker of underlying disease severity rather than a causal determinant of survival. The present analysis was not intended to suggest that distal amputation per se confers a survival benefit, but rather to describe outcome differences between clinically distinct amputation scenarios.

Although a more granular stratification of amputation levels (e.g., hemipelvectomy versus hip disarticulation or above- versus below-knee amputation) would be clinically meaningful, such subgroup analyses were not performed due to limited case numbers within individual amputation levels and the resulting risk of underpowered or misleading comparisons.

Importantly, no significant survival differences were observed between primary and secondary amputations within either group, suggesting that the timing of amputation alone has limited influence on survival once tumor-related factors and local disease control are taken into account. These findings support a flexible, patient-centered surgical strategy rather than a rigid preference for either early or delayed amputation.

### 4.5. Study Heterogeneity

This cohort comprises a heterogeneous spectrum of tumor entities, including primary bone sarcomas, soft-tissue sarcomas, metastatic disease, and a small number of skin and benign tumors, particularly in the hand and foot subgroup. This heterogeneity reflects real-world clinical practice at tertiary musculoskeletal oncology centers, where amputation is indicated across a broad range of oncological scenarios rather than confined to a single histological entity.

The primary objective of this study was therefore not to derive entity-specific prognostic estimates, but to analyze outcomes related to amputation level and timing across tumor entities. Entity-restricted survival analyses were intentionally not performed, as the limited number of events within individual histological subgroups would have resulted in underpowered and potentially misleading estimates.

Nevertheless, in individual clinical decision-making, tumor entity must always be taken into account, as biological aggressiveness and response to multimodal therapy differ substantially between sarcoma subtypes and directly influence the balance between limb preservation and oncological safety [25].

### 4.6. Limitations

This study has several limitations. Its retrospective, single-center design may introduce selection bias and limits the generalizability of the findings. Additionally, the heterogeneity of tumor entities, stages, and treatment indications represents an inherent limitation, although this was intentional to capture the full clinical spectrum of tumor-related amputations. Additionally, the long inclusion period represents a potential limitation, as advances in imaging, systemic therapy, radiotherapy, and perioperative care over time may have influenced outcomes. However, era-specific analyses were not performed due to limited event numbers and the risk of overfitting. Functional outcomes and patient-reported outcome measures were not systematically available and could therefore not be analyzed, despite their clinical relevance. Future studies should prospectively incorporate functional outcomes, prosthetic use, and patient-reported outcome measures to complement oncological endpoints in patients undergoing tumor-related amputation.

## 5. Conclusions

In conclusion, this study underscores the ongoing importance of amputation in the management of extremity tumors despite continued advances in limb-sparing techniques. Survival outcomes did not differ significantly between primary and secondary amputations, suggesting that an initial limb-sparing approach may be justified in carefully selected cases. However, achieving durable local control remains critical, as local recurrence is a strong predictor of survival. These findings highlight the need for meticulous surgical planning and reinforce the central role of multidisciplinary tumor boards in guiding individualized treatment decisions.

## Figures and Tables

**Table 1:** Patient demographics and tumor origin by cohort.

	Hands and Feet				Major				All	
	Primary		Secondary		Primary		Secondary			
Age	52		55		49		51		51	
Male	7	44%	8	57%	36	63%	29	64%	80	61%
Female	9	56%	6	43%	21	37%	16	36%	52	39%
Primary bone	5	31%	3	21%	24	42%	16	36%	48	36%
Primary soft-tissue	9	56%	3	21%	24	42%	24	53%	60	45%
Metastases	0	0%	1	8%	7	13%	5	11%	13	11%
Primary and metastases skin	2	13%	7	50%	2	3%	0	0%	11	8%
All	16		14		57		45		132	

**Table 2:** Tumor characteristics by cohorts. Staging according to UICC [22], grading according to FNCLCC [23]. Size in cm.

		Hands and Feet				Major				All	
		Primary		Secondary		Primary		Secondary			
Stage	1	0	0%	1	7%	0	0%	0	0%	1	1%
	1a	0	0%	1	7%	0	0%	0	0%	1	1%
	1b	0	0%	0	0%	0	0%	1	2%	1	1%
	2	3	19%	1	7%	2	4%	1	2%	7	5%
	2a	0	0%	1	7%	6	11%	5	11%	12	9%
	2b	0	0%	3	22%	11	18%	8	18%	22	16%
	2c	0	0%	1	7%	0	0%	0	0%	1	1%
	3	0	0%	1	7%	1	2%	0	0%	2	2%
	3a	3	19%	0	0%	10	17%	8	18%	21	16%
	3b	0	0%	0	0%	6	11%	3	7%	9	7%
	3c	2	13%	1	7%	1	2%	1	2%	5	4%
	4	2	13%	2	15%	13	22%	14	31%	31	22%
	4a	1	6%	1	7%	6	11%	3	7%	11	8%
	4b	0	0%	0	0%	1	2%	1	2%	2	2%
	not applicable	5	30%	1	7%	0	0%	0	0%	6	5%
Grading	benign	5	31%	1	7%	0	0%	0	0%	6	5%
	1	0	0%	1	7%	0	0%	1	2%	2	1%
	2	4	25%	4	29%	8	14%	9	19%	25	19%
	3	5	31%	1	7%	45	78%	32	71%	83	63%
	4	0	0%	1	7%	2	4%	2	4%	5	4%
	not applicable	2	13%	6	43%	2	4%	1	2%	11	8%
Metastases	Yes	3	19%	5	36%	24	42%	20	44%	52	39%
	- before amputation	3	19%	4	29%	17	30%	18	40%	42	32%
	No	13	81%	9	64%	33	58%	25	56%	80	61%
Size	Mean	4.4	0.6 to 13.0	2.4	0.2 to 5.5	12.2	2.0 to 31.2	11.2	1.1 to 34.5	9.9	0.2 to 34.5

**Table 3:** Level of amputation.

		Hands and Feet							Major					
		Primary		Secondary		All			Primary		Secondary		All	
Upper Extremity	Carpal	0	0%	0	0%	0	0%	Forequarter	3	5%	3	7%	6	6%
	Metacarpal	1	6%	0	0%	1	3%	Shoulder	2	4%	5	11%	7	7%
	Finger	0	0%	3	21%	3	10%	Upper Arm	5	8%	2	4%	7	7%
								Elbow	0	0%	0	0%	0	0%
								Lower Arm	2	4%	0	0%	2	2%
	All	1	6%	3	21%	4	13%	All	12	21%	10	22%	22	22%
Lower Extremity	Tarsal	6	38%	4	29%	10	33%	Hemipelvectomy	3	5%	5	11%	8	8%
	Metatarsal	3	18%	2	14%	5	17%	Hip	3	5%	6	13%	9	9%
	Toe	6	38%	5	36%	11	37%	Thigh	27	48%	16	37%	43	42%
								Knee	1	2%	2	4%	3	3%
								Lower Leg	11	19%	6	13%	17	16%
	All	15	94%	11	79%	26	87%	All	45	79%	35	78%	80	78%

**Table 4:** Indications for unplanned revision surgeries.

	Hands and Feet						Major						All	
	Primary		Secondary		All		Primary		Secondary		All			
Deep infection	0	0%	0	0%	0	0%	12	86%	9	75%	21	81%	21	75%
Delated wound healing	0	0%	0	0%	0	0%	1	7%	0	0%	1	3%	1	5%
Skip lesion	0	0%	0	0%	0	0%	1	7%	0	0%	1	3%	1	5%
Recurrence	1	100%	0	0%	1	100%	0	0	3	25	3	13%	4	15%
All unplanned revisions	1	6%	0	0%	1	3%	14	25%	12	27%	26	25%	27	20%

**Table 5:** Indications for major amputations.

	Major					
	Primary		Secondary		All	
Size	19	33%	16	36%	35	34%
Vessel/Nerve infiltration	18	31%	6	14%	22	21%
Ulceration	5	9%	5	11%	12	12%
Multiple metastases	1	2%	5	11%	6	6%
Infection	0	0%	6	14%	6	6%
Joint invasion	5	9%	0	0%	5	5%
Foot	3	5%	2	4%	5	5%
Hematoma	1	2%	3	6%	4	4%
Fracture	3	5%	0	0%	3	3%
Palliative	2	4%	0	0%	2	2%
Vascular occlusion	0	0%	1	2%	1	1%
Inadequate Osteosynthesis	0	0%	1	2%	1	1%

**Table 6:** Multivariable Cox proportional hazards regression analysis for overall survival.

	Hazard Ratio	95% Confidence Interval	*p*
Local recurrence	2.21	1.01–4.85	0.048
Major vs. hand/foot	0.58	0.21–1.59	0.287
Age	1.00	0.99–1.02	0.811
Metastases	1.65	0.86–3.16	0.136
Tumor size	1.03	0.99–1.08	0.115
Primary vs. secondary	1.13	0.60–2.13	0.710

## Data Availability

The original contributions presented in this study are included in the article. Further inquiries can be directed to the corresponding author.

## Abbreviations

The following abbreviations are used in this manuscript:

CI	Confidence Interval
FNCLCC	Fédération Nationale des Centres de Lutte Contre le Cancer
LSS	Limb-Sparing Surgery
LR	Local Recurrence
OS	Overall Survival
R0	Microscopically negative resection margin
R1	Microscopically positive resection margin
UICC	Union for International Cancer Control
